# The Futures of Europe: Society 5.0 and Industry 5.0 as Driving Forces of Future Universities

**DOI:** 10.1007/s13132-021-00854-2

**Published:** 2022-01-05

**Authors:** Elias G. Carayannis, Joanna Morawska-Jancelewicz

**Affiliations:** 1 School of Business, Department of Information Systems and Technology Management, George Washington University, Washington, DC USA; 2 Faculty of Human Geography and Planning, Adam Mickiewicz University, Poznan, Poland

**Keywords:** Quadruple/Quintuple Helix Model, Digital (social innovation), Society 5.0, Digital transformation

## Abstract

The concept of Society 5.0 and Industry 5.0 is not a simple chronological continuation or alternative to Industry 4.0 paradigm. Society 5.0 aims to place human beings at the midpoint of innovation, exploiting the impact of technology and Industry 4.0 results with the technological integration to improve quality of life, social responsibility and sustainability. This ground-breaking perspective has common points with the objectives of the United Nations Sustainable Development Goals. It also has major implication for universities transformations. Universities are called upon producing knowledge for new technologies and social innovation. In our paper, we argue that digitalisation opens new perspectives for universities and can become one of the main drivers of their change. Incorporating the assumptions of Society 5.0 and Industry 5.0 into the universities practices and policies will allow both universities and societies to fully benefit from digital transformation. Making the human-oriented innovation as the universities trademark and developing new cooperative models will also help to achieve sustainable priorities. The use of the Quintuple Helix Model (QHM) might foster the process of necessary transformations capacities as it integrates different perspectives and sets the stage for sustainability priorities and considerations. As far as the practical goal is concerned, the paper proposes a set of recommendations for universities aiming at developing new forms and channels of distribution of education, research and innovation within in the context of QHM and Society 5.0. We call them socially and digitally engaged model.

## Introduction

“Universities are created to tackle the unknown. While their future cannot be planned, the tools they have at their disposal to meet the future can be improved.”^1^

The recent COVID-19 pandemic has undoubtedly raised new questions regarding the future image of the innovation ecosystems, the relations between the main actors of innovation and the challenges they need to face in order to rapidly transform to new modes of operation related to digitalisation, digital transformation (DT) and to become resilient organisations. This time of global crisis has also accelerated a world-wide debate on related wicked and complex problems and challenges called Sustainable Development Goals (initiated by United Nations decades ago and proposed in 2015 as Agenda 2030) that gained their momentum. “Green” and “Digital” have become “big ideas” and leitmotivs of this debate. Digitalisation and greening of economy are considered as twin concepts that promote sustainable development. They go in hand as for example new digital technologies including artificial intelligence (AI) are used to collect, assess, analyse data and communicate those results to a wider public. They help to raise awareness and to advocate for more engagement form the society in solving complex problems through, e.g., citizen science projects. They lead to a better governance and more inclusive innovation processes. There are many challenges related to DT such as data security and transparency, or the carbon footprint of ICT sector, but still, there is a wide agreement on digital and green alignment and interrelation. Since there is no “turning back” as the digital and green transitions are the facts, the discussion about future is needed in order to be prepared for the next crisis but also to adjust and adapt to new disruptive developments such as AI and other new and key-enabled technologies like biotech, nanotech, Internet of Things (IoT) and robotics, cloud computing and machine learning. They are bringing significant changes to economy and society and undoubtedly we can say that the world has been undergoing substantial transformation with innovation as driving force. The 2030 Agenda also express high expectations that innovation will play a central role in addressing SDGs.

In this article, we will use AI for describing a wide range of technologies associated with fourth industrial revolution, as proposed by Procter et al. (2020, p.5):

[AI is] an umbrella term to cover a set of complementary techniques that have developed from statistics, computer science and cognitive psychology. While recognising distinctions between specific technologies and terms (e.g., artificial intelligence vs. machine learning, machine learning vs. deep learning), it is useful to see these technologies as a group, when considering how to support development and use of them.

We also aim to focus on opportunities arising from AI and digital and green transitions and reflect on new considerations and approaches that are more balanced, sustainability oriented and human-centric. We therefore concentrate on theoretical views and considerations related to the present/future concept of Industry 5.0 and Society 5.0 and their potential to generate new values to economy, society and the natural environment and to build new system of (eco)innovation that promotes in a systemic way open, social, digital, technical innovations for the benefit of people. In our understanding, this process is possible within Quadruple/Quintuple Helix Models of Innovations (Q2HM) where the universities as drivers of knowledge and the anchors of innovation play a crucial role in orchestrating the process of innovation and are pursuing the change (Goddard et al., 2016).

In this article, we also refer to the concept of Mazzucato (2018) that calls for mission-oriented innovation, social innovation that are cross-disciplinary, cross-sectoral and cross-actor innovation with important role of citizens as active participants of innovation process. Research and innovation missions should thus aim to improve society’s welfare.

We agree with Mazzucato that *Openness and collaboration are not a nice complement, but rather a critical factor for Success* (Mazzucato, 2018, p.5).

We also claim that (digital) social innovation (DSI) can be a useful tool for supporting green and digital transition of universities since they lead to transformative change and are social in their ends and means, remaining open to the territorial, cultural, etc. variations they might take (Guide to Social Innovation, 2013). We propose the model of socially and digitally engaged university that embraces new university roles in ecosystem of innovation. The ecosystem comprises a multilayer framework in which institutions interconnect to develop and share information and knowledge required for the development of new innovation processes (Costa & Matias 2020, p. 2). In this model, the universities are envisioned as prototyping places for social and digital transformations (SDT) and creating power capital. The last we understand as having two dimensions. First, refers to a strong academic leadership that recognises the value of diverse networks which extend beyond their zones of proximity, familiarity and competence; base on a dialogue and influence. We also think that power capital reflects the power of scientist and students to become change agents. We agree with Blewitt (2010, p. 396) that claims: *with information growing by the second, knowledge expanding exponentially and wisdom still in short supply, applying new digital technologies to the sustainability imperative, requires a transdisciplinary synthesising mind and a higher educational specialism that helps students to become generalists*. Second dimension of power capital reflects the engaged and inclusive society, playing an active role in innovation ecosystem. We might call it Super Smart Society in Society 5.0, where value is generated not from clusters of tangible assets but rather from knowledge spaces—spaces where data and information are gathered and then deciphered and deployed through knowledge (Society 5.0 A People-centric Super-smart Society 2018 p. 11).

The recently discussed concept of Society 5.0 (S5.0) and Industry 5.0 (I5.0) (Carayannis et al., 2021a, b, c; Carayannis & Morawska-Jancelewicz, 2021; Carayannis, 2021a, b, c, d; Carayannis, 2020b; Breque et al, 2021; Fukuyama, 2018) highlights the need to re-think existing working methods and approaches towards innovation and to focus them on developing human-oriented solutions and social innovation (Morawska-Jancelewicz, 2021). Society 5.0 and Industry 5.0 both reflect fundamental shift of societies and economies towards new paradigm to balance economic development with the resolution of social and environmental problems and to tackle challenges associated with human–machine interactions and skills matching (Breque et al., 2021). In this new paradigm, the importance of knowledge is not determined exclusively by competitiveness and productivity, but by taking into account the creation of social well-being, the impact on the quality of life and co-creation of knowledge as part of public–private partnerships (Morawska-Jancelewicz, 2021 p. 3). It also stresses that *even the most advanced technology should not be above humanity* (Sułkowski et al., 2021).

This paper attempts to address the gap of relatively few studies on institutional change and incentive structures that influence the ability of universities to engage in (digital) social innovation within digital and green transitions and fills the gap of identifying connection between Society 5.0 and Industry 5.0 concepts and the Q2HM framework. We believe that universities should take strategic measures and build comprehensive programmes and models of cooperation with society within new growing challenge of digital and green transitions. We propose to adapt the socially and digitally engaged university model that could be a tool for stimulating and strengthening their functions within a modern regional innovation system allowing for an active role in addressing global challenges including SDGs.

The organisation of the paper is designed in the following way. Chapter 1 focuses on recent discussion related to a future vision of Industry 5.0 and Society 5.0. In particular, it explains how these concepts are related to present innovation ecosystem and the new paradigm of human-centric innovation. Chapter 2 focuses on Quadruple/Quintuple Helix Model as a framework for co-evolution of knowledge-driven economy. Chapter 3 discusses the concept of social and digital social innovation and examines what implications it might have as real driver of social change, thus promoting also equality and shared prosperity. Chapter 4 summarises the challenges universities face on the road to digital and green transitions. Chapter 5 presents a socially and digitally engaged model as a future vision of a university in I5.0 and S5.0. The conclusions drawn therefrom may add value to the ongoing scientific discourse on the development of (digital) social innovation by universities and their importance for the innovative growth of regions within Q2HM in I5.0 and S5.0. Hence, this article offers a primer on I5.0 and S5.0 by discussing the key definitions and their implicit meanings for the future university model.

## Industry and Society 5.0

Industry 5.0 can be considered as the answer to the demand of a renewed human-centred/human-centric industrial paradigm, starting from the (structural, organisational, managerial, knowledge-based, philosophical and cultural) reorganisation of the production processes to then generate positive implications first within the business perspectives and secondly towards all the components belonging to the innovation ecosystem (Carayannis et al., 2021c, p. 5; Carayannis 2021a, b, c, d). Industry 5.0 relies on three core elements: human-centricity, sustainability and resilience. If industry should become the provider of true prosperity, the definition of its true purpose must include social, environmental and societal considerations (Breque et al., 2021, p. 15).

Society 5.0 (Super Smart Society) is a new guiding principle for innovation. It promotes convergence between cyberspace and physical space *enabling AI-based on big data and robots to perform or support as an agent the work and adjustments that humans have done up to now* (Fukuyama, 2018)*.* As stated in Cabinet Office of Japan:

Society 5.0 takes systemic approach but focusing on human beings with the aim to involve a wide variety of actors that in the past have only participated in non-visible ways (e.g. women and young people). It is a space for accommodating various bottom-up ideas.

Society 5.0 calls for “systemization” of services and projects, more advanced systems, and coordination between multiple systems – thus aiming to serve as a Smart Bridge between the techno-centric and human-centric perspectives.

Society 5.0 states that companies, universities, and others who are responsible for innovation systems should strengthen their cooperation, breaking down organizational walls and promoting open innovation.

What should be strengthened is that Society 5.0 considers social capital as its key asset and it promotes globally targeted open innovation with human-centric priorities. In this concept, every citizen representing each generation has a role to play in innovation process. The Super Smart Society is build upon delivering the concrete, targeted and personalised, just on-/in-time solution to the people with the aim to provide healthy and safe environment and to promote people well-being. It is still a vision, directive or goal and not the reality. Yet, it opens new perspective to understanding and utilising the technological advancement and digital transformation for the benefit of society. *Society 5.0 is a kind of bond between changes taking place in the technology, digital, and information flow areas and focuses its activities on the concept of sustainable development of societies* (Sułkowski et al., 2021, p. 4). In other words, the vision of Society 5.0 requires us to think about two kinds of relationships: the relationship between technology and society and the technology-mediated relationship between individuals and society (Society 5.0 A People-centric Super-smart Society, 2018 p. 5). It also leads to new types of relation between green and digital.

Society 5.0 and Industry 5.0 represent the convergence with Q2HM but the question remains how we can drive our efforts to address relevant social challenges within socio-economic activity? How we can build those smart bridges between techno- and human-centric orientation in innovation? How to mitigate and buffer disruptions of societies and economies or how to minimise them? In our vision, we call for a systemic approach to achieve that within Q2HM and to accelerate digital and green transformations through (digital) social innovation. *In designing this transformation, universities can function as core bases of value creation, and become places where transformation is prototyped with the cooperation of multiple stakeholders* (Hamaguchi, 2020, p. 104).

### Towards Smart Sustainability Within Quadruple/Quintuple Helix Models

The literature examination shows the evolution of contemporary mechanisms of creating innovation by taking into account their network and bottom-up nature, which is departing from the conception of the triad universities-business-state to the model of the Q2HM universities-business-state-civil society institutions, in which an active participation of citizens is becoming increasingly important (Caryannis & Rakhmatullin, 2014). “Tailor-made” solutions with the active participation of interested parties are a significant feature of social innovation. They allow to form a more inclusive, democratic innovation system based on a dialogue and reflecting also values of the society. They also emphasise the importance of knowledge in creation of social well-being and in general the quality of life. Cross-sector or multi-actor collaboration could significantly contribute to a local or regional transition to sustainability (Morawska-Jancelewicz, 2021).

Quadruple and Quintuple Innovation Helix frameworks can serve as architects for a better future and emphasise that, over the medium to long term, true and transparent democracy constitutes a sine qua non for smart, sustainable and inclusive growth (Carayannis et al., 2021a, p. 11; Carayannis, 2017; Carayannis, 2021d).

**Fig. 1:**
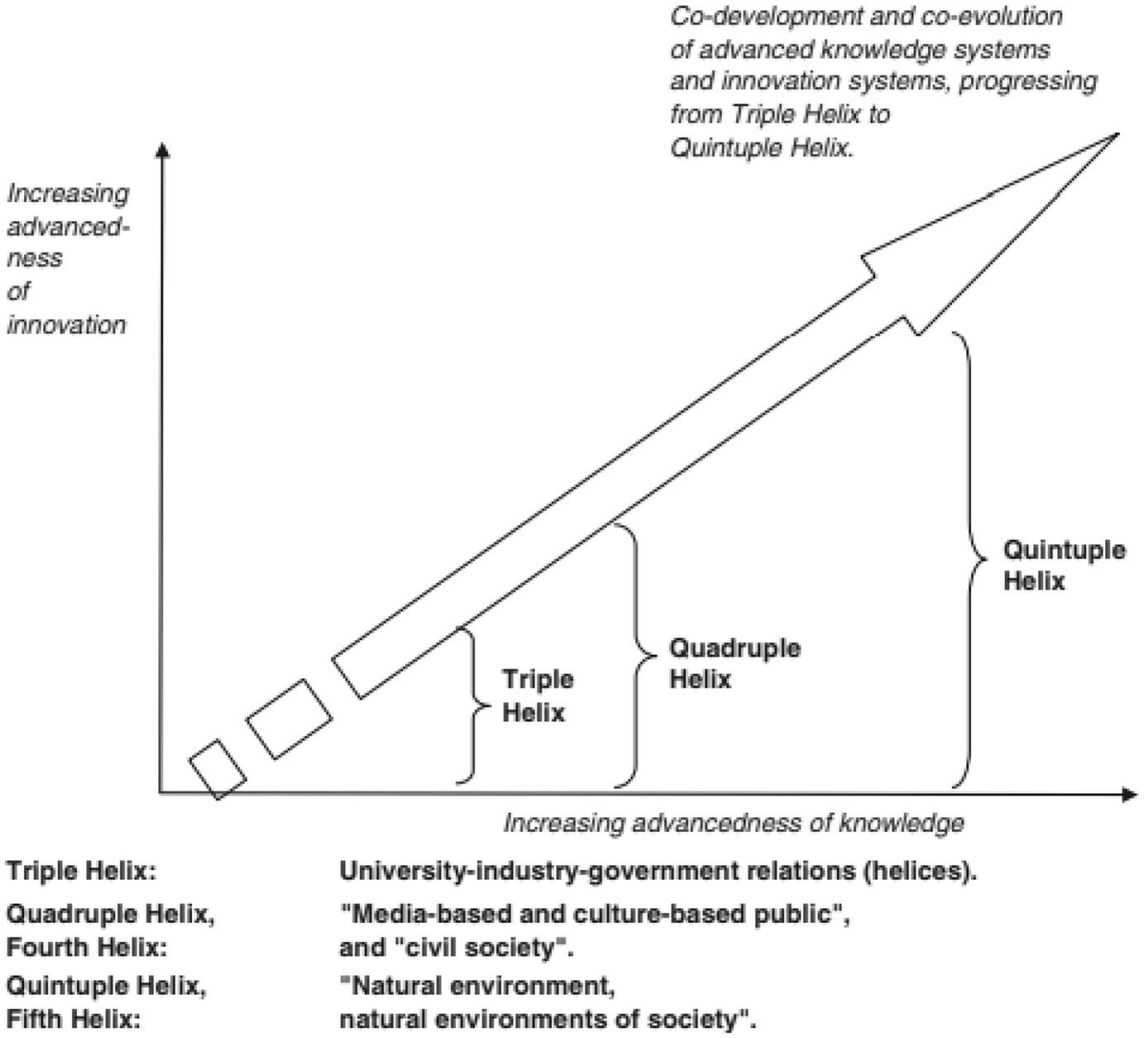
Co-development and co-evolution of advanced knowledge production. Source: Carayannis & Campbell, 2011 p. 343

The concept of the Q2HM asserts that an advanced knowledge democracy is actually necessary to advance knowledge and innovation (Fig. 1). To some extent, democracy is “doomed” to succeed in approaching the ecological issues and, again, it may only be democracy, finally creating and generating the knowledge and innovation, which is necessary to solve the ecological challenges for a new future. Educational organisations and higher education institutions (for example, universities) can be expected to play a crucial role. Democracy, environmentalism and the innovation-driven knowledge economy may be co-evolving (Carayannis 2020; Carayannis et al., 2021a, p. 12; Carayannis, 2021a, b, c, d).

Since innovation is perceived as a vital factor for economic and social development of organisations, regions and countries, it represents a mean for economic growth, productivity increase, knowledge creation, new occupations and wealth proliferation. Innovation is also a mean by which organisations seek to renew their management skills in particularly complex environments. Today’s economy is characterised by knowledge-intensive activities that contribute to an accelerated pace of technical and scientific advance, as well as rapid obsolescence; thus, the ability to manage complexity and uncertainty is not achieved through their negation. In this sense, innovation and knowledge in smart environments should be the result of a sharing process that involves all the actors of an innovation ecosystem, interpreting complexity as an opportunity and not as a threat. This type of “openness” fits well with the new logics of Industry 5.0, according to which human-centred solutions should be guaranteed for systemic and sustainable development (Carayannis et al., 2021a, b, c).

In this new innovation system logic, the environment should be regarded as an active partner of innovation, not a resource to be exploited. The need for a more sustainable development and climate change challenges are reflected in Q2HM that adds the fifth dimension — the environment, and sets the stage for sustainability priorities and considerations so that nature is central and equivalent component of and for knowledge production and innovation (Carayannis et al., 2020; Carayannis, 2017). The Quintuple Helix framework, however, sees and interprets environmental and ecological problems also as opportunities by identifying them as possible drivers for the production of future knowledge and the creation of future innovations (Carayannis & Campblell, 2011). The Q2HM models are seen as playing an important role in fostering the shift from technical to social innovations and especially towards a more balanced human-centric and techno-centric approach (Table 1).

**Table 1:** Evolution of techno- and human-centric innovation

	**Industry 4.0**	**Society 5.0**	**Industry 5.0**
Triple Helix	Techno-centric		
Quadruple Helix		Human-centric	
Quintuple Helix			**Balanced techno- and human-centric**

Source: Authors

### (Digital) Social Innovation

Social innovation is based on the assumptions of open innovations and is part of the conception of regional endogenous development, where a key factor of development is people with their knowledge and skills. The progress is assumed to be endogenous in character and the role of a particular factor leading to an increase in the productivity of other resources is ascribed to human capital and knowledge. This conception emphasises the noneconomic nature of development factors. It also points out that innovations result to a great extent from the accumulation of the experiences of a given actor (mainly through learning by doing) and from continuous learning through interaction (learning by interaction). This leads to an improvement in skills and qualifications, and contributes to increased labour productivity and technological advancement. The accumulation of knowledge in a specific space, along with learning mechanisms, makes it possible to create and disseminate new knowledge resources and innovation processes (Morawska-Jancelewicz, 2021).

Innovations appear as a result of cooperative activities, and networks, clusters or common values and interests are keys to their creation. In the traditional approach, companies manage innovations through licences, patents issued by certified agencies. In the new approach, local and regional networks as well as mutual trust, and also intermediary bodies bringing together organisations from different sectors, cultures, industries and specialisations are equally important. Relationships between partners determine the success of the innovation process as well as its efficiency and the effective management of the public means financing it.

However, social innovations represent a very wide range of activities, embrace various sectors and fit into a wide spectrum of scientific disciplines, and they are developed and proposed by various types of organisations, interested parties, teams or individuals. Advanced IT tools, AI and new technologies are increasingly often used to implement them. The priority objective of social innovations is social change. They are determined by an additional motive, i.e., a social mission and social added value. It does not mean depriving them of economic viability. Yet, the underlying purpose of innovative ideas should be the solution to a specific problem and a change for the better. They often require an interdisciplinary approach combining frequently remote fields of knowledge and are created by teams of people representing different organisations and competences (Morawska-Jancelewicz, 2021).

**Fig. 2:**
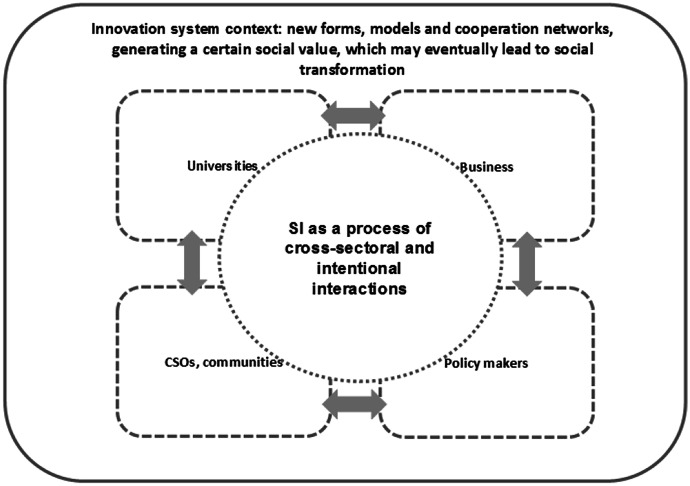
Social innovation as a process. Source: Morawska-Jancelewicz, 2021

Social innovations are innovative solutions that have a predefined social objective, are used to meet specific social needs, lead to the development and strengthening of civic society, and are based on cross-sectoral and interarea cooperation between actors, thereby also changing social relations. Following this definition, we propose to consider social innovation in two ways: as a process and as an output of innovative activity. In the first case, we can say that *social innovation is a total of intentional, responsible interactions of various interested parties, who share the common objective of finding a solution to a specific social problem* (Fig. 2).

There appear new forms, models and cooperation networks, most often within a given area, generating a certain social value, which may eventually lead to social transformation. The idea of fair-trade, participatory budget, living lab concepts, a new care system for dependent persons or home schooling can be examples of social innovation as a process.

**Fig. 3:**
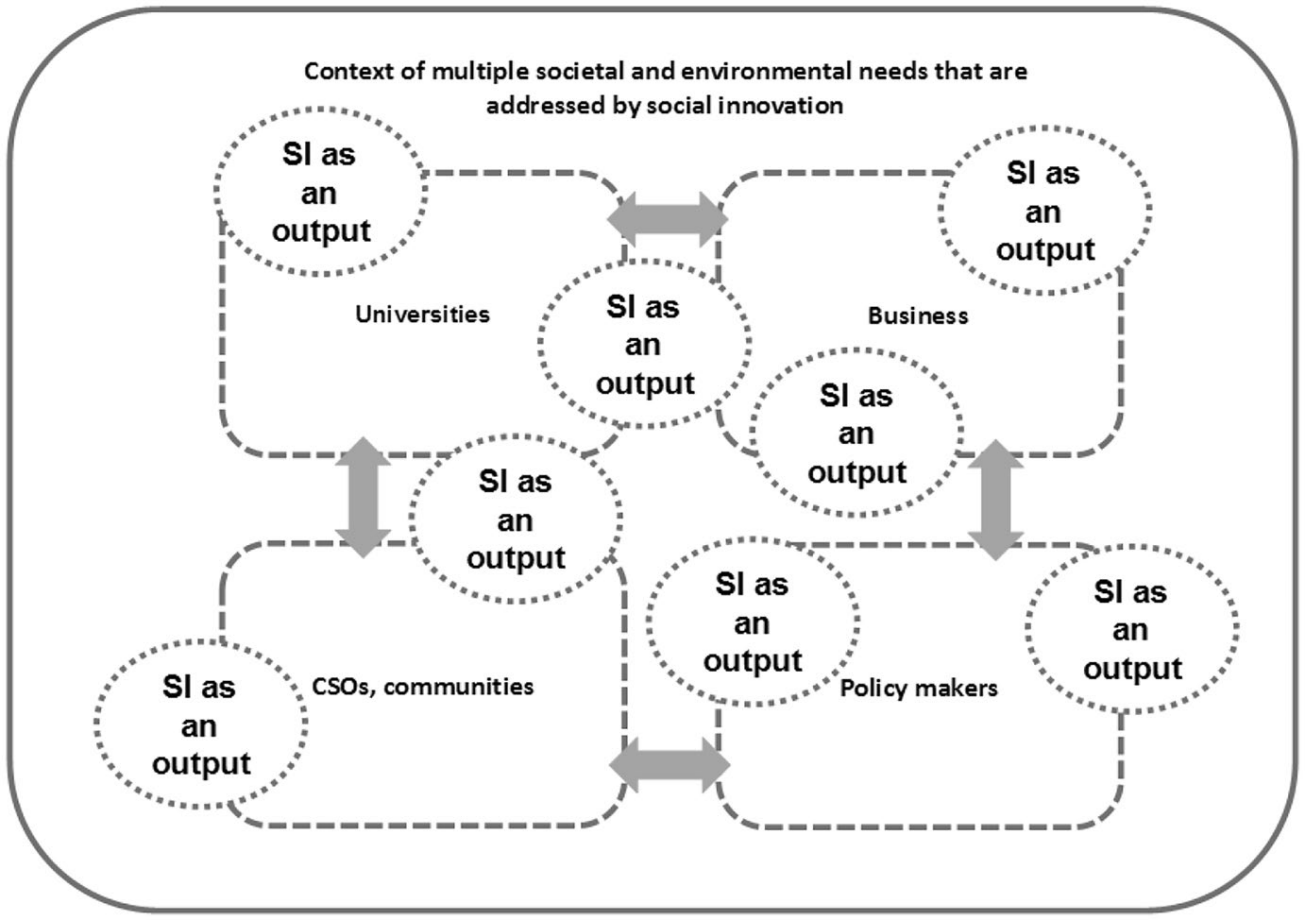
Social innovation as an output. Source: Morawska-Jancelewicz, 2021

In the case of the social innovation defined as an output, *there are new or more effective solutions to a specific social problem, created within interdisciplinary and cross-sectoral cooperation, which are environmentally friendly and support social development* (Fig. 3). An example of this type of innovation will be certain products, services, (digital) tools which improve the quality of life, e.g., software for the blind and partially sighted, specific education programmes for excluded groups, e.g., children from rural areas, and intelligent transport adapted for various passengers (Morawska-Jancelewicz, 2021).

Misuraca & Pasi (2019) emphasise that the potential of social innovation is further increased by the availability and accessibility of new emerging tool and technologies like AI. This IT-enabled or digital social innovation can help to digitalise social services process and to making them more proactive, and more goal and needs-driven. They also promote direct engagement of citizens in the whole social services process design and management. In other words, (digital) social innovation can become the real driver of social change, thus promoting also equality and shared prosperity.

The similar approach can be found in Bria et al. (2015) who see the civic role of digital technologies in helping to mobilise large communities, share resources and empower people. The following proposed definition of DSI clearly reflects its core relevance:

(…) we define DSI as “a type of social and collaborative innovation in which innovators, users and communities collaborate using digital technologies to co-create knowledge and solutions for a wide range of social needs and at a scale and speed that was unimaginable before the rise of the Internet” (Bria et al., 2015, p. 9).

According to Serpa and Ferreira, (2019, p.11–12), DSI emphasises the intersection of three elements: the innovation process, the social world and the digital ecosystem. In their understanding, the technology is not the key element. It is rather the tool or a mean to address social challenges. The social dimension reflects the view that the focus of innovation is not the technology in itself. DSI delivers more effective, sustainable and ethically adequate solutions thanks to integrating those three dimensions and offering more target innovation. Their potential and range is still growing. The recent European Commission (Foresight), (Warnke et al., 2019, p. 256-262) emphasises the development of Rapid Social Innovation Breakthroughs (RSBs), which indicates that they are relatively new and potentially disruptive, but still already practiced by the society and becoming more relevant in achieving goals of European Research and Innovation Policy. Just to name a few, they are: collaborative innovation spaces; access/common based economy; read/write culture; reinvented education; body 2.0.; the quantified self; car-free city; new journalist networks; local food circles; alternative currencies; owning and sharing health data; basic income and life catching (Table 2). A large proportion of RSBs has strong links to AI or other IT technologies and it is assumed that by 2038, a strong wave of AI-based innovation related to for example biotechnology, health and sustainability will bring both significant potentials and risks at the same time. Examples include neuromorphic chips, bioplastic, 4D printing or local food circles. It is crucial to ensure that suitable framework conditions and accompanying social innovations are in place in good time. It is very different to predict what kind of impact they will bring. Some might turn out to be more disruptive than expected (Warnke et al., 2019).

**Table 2:** Examples of contemporary Rapid Social Innovation Breakthroughs (RSBs)

Categories of RSBs	Examples
Collaborative innovation spaces	Makerspaces, hackerspaces or innovation labs, communal workshops, where people can share ideas and tools
Gamification	The application of game-design elements and game principles in non-game contexts to improve user engagement, organisational productivity, flow, learning, crowdsourcing, employee recruitment and evaluation, ease of use, usefulness of systems, physical exercise, traffic Violations, voter apathy and more; data generation combined with participation via gaming; citizen science; gaming for physical education and health
Access/commons-based economy	Sharing economy, access economy; new forms of organising access to good and services; online sharing e.g. TripAdvisor; non-profit tool libraries and freecycling; commons, e.g., resources shared by a restricted group of people following shared rules like for example a lake that is jointly managed by a community of villagers; commons-based peer production like Wikipedia, Linux and TripAdvisor; goods and services are traded based on access rather than ownership, e.g., ZipCar and Airbnb
Read/write culture: diversifying information gatekeepers	Through “social media,” people become “active audiences” with the ability to not only share but also generate, manipulate and transform digital content, e.g., vloggers
Reinventing education	A huge diversification of education providers, apps and of training partnerships; new knowledge providers; MOOCS; flexible learning, peer to peer platforms
Body 2.0 and the quantified self	The permanent monitoring of the human body and the almost medical monitoring of one’s bodily functions, via wearables, Smartphone Apps or separate sensors. This new social movement encourages users to better understand themselves by collecting data on every aspect of their daily life: from food consumption, air quality, blood oxygen levels, arousal, to bowel movements and so on. It has also become present in some companies monitoring their workers
Car-free city	Car-free cities greatly reduce petroleum dependency, air pollution, greenhouse gas emissions, automobile crashes, noise pollution and traffic congestion. The innovation is to transform highly car-dependent cities or construct new car-free cities from scratch; 15-min city
New journalist networks	Journalists work together on specific targets to reveal news and find evidence for big or small but global stories. They co-operate globally — with newspaper journalists or freelancers
Local food circles	A new way of conceiving of and organising our agricultural and food systems. It links the many people involved in food production together in interdependent, holistic ways; promoting the consumption of safe, regionally grown food that will encourage sustainable agriculture and help to maintain farmers, who will sustain rural areas; slow food, organic food, indoor (hydroponic) gardening, community gardening, permanent agriculture
Owning and sharing health data	There are movements to create spaces, in which the persons who are the subjects of the data know that their data are safe and can be used, e.g., for research, and in which individuals benefit directly from providing their data. This is a counter-initiative against companies exploiting personal health data generated in different situations, intentionally and unintentionally
Alternative currencies	Crypto-currencies (digital) and non-digital; cashless society
Basic income	Guaranteed minimum income (GMI) or “basic income” is a system of social welfare provision that guarantees all citizens or families to have an income sufficient to live on
Life catching	Means collecting, storing and displaying one’s entire life for private use, or for friends, family, even the entire world to peruse, e.g., scrapbooks. Millions of people are digitally indexing their thoughts, rants, pictures, video clips; most of them with new means online, disclosing the virtual caches of their daily lives, exciting or boring. The purpose of life caching is mainly keeping the memory

Sources: Authors on the basis of European Commission (Foresight) (Warnke et al., 2019)

### Digital Transformation and Greening the Universities

Innovation mechanisms are diversifying and lead to new business models, based on digitised information. In the future, innovative information-based technologies such as the IoT, AI and robotics are expected to generate new added value (Cabinet Office of Japan).

The expression “digital transformation” (DT) has called the attention of organisations from all sectors around the world (Rego et al., 2021). It attempts to measure the extent to which an organisation is able to benefit from the use of information technologies (IT), but it is also seen as an evolutionary process through which IT becomes a fundamental element of its daily life, affecting all dimensions that involve both people and the organisation itself (Rodríguez-Abitia & Bribiesca-Correa, 2021, p. 3). Integrating and exploring new digital technologies is one of the biggest challenges that all organisations currently face. No sector is immune to the effects of DT, including higher education. As Rego et al. (2021) argue following recent study by Vial (2019), digital transformation is a multidimensional phenomenon, levered by technology, which impacts society, politics and economics. Digital transformation means development, in the sense of integrating not only machines and IT infrastructure but also people. It calls for a reinvention of an organisation—its vision and strategy, organisation structure, processes, capabilities and culture. Artificial intelligence can be both enabler and threat to organisations so that organisations must find their own ways to successfully manage their transition towards desired future. A great variety of definitions of DT exist in the literature (Abad-Segura et al., 2020; Almaraz et al., 2016; Breque et al., 2021; Carayannis et al., 2021a, b, c; Carayannis & Morawska-Jancelewicz, 2021; Carayannis, 2021a, b, c, d; Carayannis, 2020b; Carayannis, 2019; Fukuyama, 2018; Hashim et al., 2021; Giang et al., 2021; Rego et al., 2021; Rodríguez-Abitia & Bribiesca-Correa 2021; Sułkowski et al., 2021; Vial, 2019; Vishnevsky et al., 2021) that stress the role of the contextual factors, the size or location of the organisation and its cultural, regional embeddedness.

In order to take advantage of emerging technologies and their rapid expansion in human activities, organizations must reinvent themselves and transform all their processes. For this reason, DT requires a change of focus and involves innovating in technology and modifying the institutional culture to guarantee the evolution of DT (Abad-Segura et al., 2020, p.5). This is also true for universities. Rodríguez-Abitia & Bribiesca-Correa (2021) propose a framework to assess the level of digital maturity in universities (Table 3).

**Table 3:** Digital maturity of universities

Dimensions of digital maturity	Tools
The ability to provide an appropriate IT infrastructure	e.g., network connectivity, computing devices in labs or loan systems, equipped classrooms
The ability to apply technology to the teaching and learning process	e.g., open educational resources, interactive lessons, artificial intelligence and robotics, 3D platforms, repositories and virtual simulators
The ability to provide collaboration and organisational platforms to integrate processes and people	e.g., workflow systems, educational social networks, learning management systems integrated with academic administration systems, and virtual communities

Source: Rodríguez-Abitia and Bribiesca-Correa (2021, p.5)

Hashim et al. (2021) argue that DT has brought *a window of opportunity for universities* empowering students and academics. Independent online-based learning system/mechanism inspires learners to master various relevant disciplines to enter the work field. Specifically, it deals with (a) learning content, (b) technical know-how and (c) other competencies. It has provided digital but internet-driven solutions for the entire element of the value chain, thus universities can be exceptions here. This process has not only generated unique and innovative business models worldwide, but the universities have become increasingly entrepreneurial. *The utilization and integration of digital technologies enable universities to go beyond their conventional virtual borders, influencing the portfolio of courses, regulating the delivery model and the entire value chain of a university* (Hashim et al., 2021). Currently, the adoption of technologies by universities is related to a paradigm shift, where technology is conceived as a complex and interconnected environment that enables digital learning (Abad-Segura et al., 2020 p. 1).

*The digital transformation is a long-term process, going through many stages, requiring many resources to participate and the support of regulatory agencies, institutions, and policies. As a result, several questions that relate to the effectiveness of digital transformation as well as the fundamental factors that should be considered when participating in the digital transformation process, have been increasingly emerging. Therefore, the assessment of readiness for the necessary changes to adopt digital transformation in higher education institutions is a demanding goal* (Giang et al., 2021 p. 5).

We understand the greening of universities as *increasing awareness and taking concrete action towards a green, environmentally-friendly and resource-efficient university. This may address the university’s mission and campus, and its members, but also entails a contribution towards its larger community and surroundings. It may or may not be part of a broader approach to address the SDGs and contribute to the 2030 Agenda* (Greening in European higher education institutions 2021 p. 3). The EUA study revels that universities address sustainability through a large range and diverse measures of activities. It is also the truth for the Green Metrics University Rankings or Times Higher Education Impact Ranking. Despite the criticism they cause (due to, e.g., questionable methodologies), they refer to all universities missions as well as to their strategic policies. The examples are green mobility (measures to change travel habits of students and employees); extra curricula policy or study programmes dedicated to sustainability; fostering the green use of shared research infrastructures or reducing the environmental footprint of laboratory research; recycling and waste management, sustainable construction and renovation and the use of resources; new partnerships end engaging with different organisations in green activities; contributing to local, regional and national debates; involvement in sustainable university networks and alliances; and linking their policies and strategies to Agenda 2030 or EU Green Deal.

### The Role and Future of Universities in Industry and Society 5.0

Since the digital and green transitions are interlinked, they pose both a challenge and opportunity for universities. Universities need to find a *sustainable equilibrium between ecological, economic and social concerns, navigating the digital transition and dealing with (geo)political uncertainty* (Jørgensen & Claeys-Kulik, 2021 p. 10). The universities are called to *strongholds and billboards to democracy.* Navigating between academic autonomy and freedom and societal and political expectation requires from universities to build strong and resilient structures and policies and to reflect about their future strategically. In this article, we advocate for universities to take opportunities coming from green and digital transitions and to create a thriving and innovative ecosystem integrating and using both physical and virtual spaces for accommodating to societal needs. We think that universities need to *move beyond the future.* They have the tools, they just need to use them. In line with Van den Akker (2017) statement: universities are encourage to fully embrace the societal impact agenda, to engage with others across the broad spectrum of the research ecosystem; to develop open, explicit and transparent reward systems that include the value of all kinds of impact and to continuously seek to support and promote societal impact as a dynamic, open and networked process in a culture of sustained engagement and co-production of knowledge (Morawska-Jancelewicz, 2021).

Universities role in orchestrating multi-actor innovation networks in a non-linear innovation process requires a systemic approach to innovation, that is challenge-driven, collaborative and interdisciplinary. It should be integrated throughout all university missions including third and fourth missions, leading to a deeper cultural project of creating co-creation innovation spaces (The Role of Universities in Regional Innovation Ecosystems, EUA, 2019 p. 10). The fourth mission concept is particularly relevant as it puts emphasis on the universities roles in sustainable development and we understand as the co-creation with stakeholders and communities for sustainability. Riviezzo et al. (2019, p. 31) propose a term fourth mission as “the promotion of social, cultural and economic development of the host community, that, in a very broad sense, leads to argue that university should contribute also to the quality of life perceived by the community itself” (Morawska-Jancelewicz, 2021). We believe that integrating both green and digital transition in the university missions leads to the development (digital) social innovation and to the more open and human-centric innovation ecosystem (Table 4).

In this paper, we agree with Grau et al. (2017) who notice that universities may fulfil a key role with regard to solving (a) global problems, the reflection of which is the 17 Sustainable Development Goals of the global UN agenda, and (b) local problems: social, cultural and economic, contributing to better development and competitiveness. The dualism of activities results from contemporary expectations and development trends manifested, e.g., by the promotion of social innovation, scientific social responsibility, or the conception of Responsible Research and Innovation (Morawska-Jancelewicz, 2021).

**Fig. 4:**
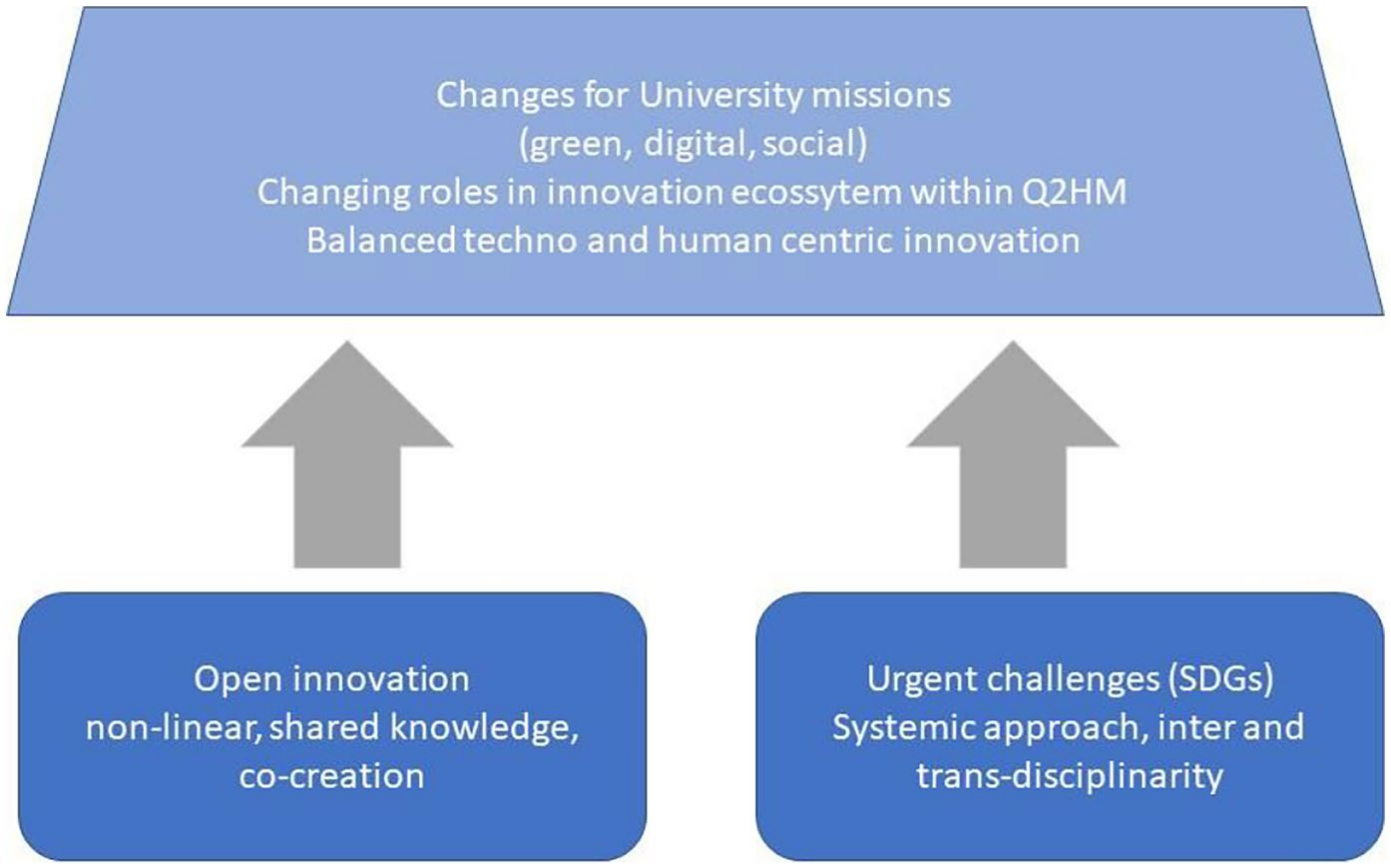
Innovation and the changing role of universities. Source: Authors based on The Role of Universities in Regional Innovation Ecosystems, EUA, 2019 p. 10

We assume that the function and role of university and its employees in the (digital) social innovation process is based on three pillars: (1) A university provides knowledge (existing or developed as part of the cooperation with the environment) which supports the creation of innovation. (2) A university shares its tangible and intangible assets. (3) A university supports (digital) social innovation development by advising social innovators and involving interested parties. Knowledge, support resources may be provided at various stages of creating social innovation and in different dimensions (Benneworth & Cunha, 2015, p. 10–12) (Fig. 4).

Responding to digital and green transformations requires new future-oriented strategies and organisational change that promotes the understanding and embracing digital culture. In this sense, DT is a change not only in technology but also in about people and organisational structures (Almaraz et al. 2016). One of the key challenges is to percolate the digital enthusiasm and willingness from the senior leadership to middle management and operational levels across university. Universities need to build and respond to a new mindset embracing new technologies and the new concept of green university campus. Just to list a few concrete challenges that ought to be addressed immediately: mobile-based AI-powered software for research and education; online backup programmes, new tech infrastructure, new managerial skills related to DT, re-skilling and up-skilling, digital work experience, digital literacy and digital exclusion/inclusion and assessment of the digital learning outcomes. These are not simply buzz words but real challenges that need to be addressed.

Almaraz et al. (2016) propose an interesting approach to assessing DT of universities and focus on seven dimensions of DT (Table 5). This analytical framework assumes various dependencies between different levels of DT analysis and interlinks and inter-applications (e.g., online learning contents and IT infrastructure).

**Table 4:** Developing (digital) social innovation within university missions in the context of green and digital transitions in Quadruple/Quintuple Helix frameworks

Analysis level	Variables
University campus	Mobility around campus
	Sustainability of the university campus
IT infrastructure	Infrastructure for information processing
	Communications infrastructure
Administration	Automatisation of university management processes
	Digitalisation of the user experience
	Impact on interactions with the environment
Teaching	Face-to-face teaching
	Online teaching
	Teaching innovation
Research and knowledge transfer	Digital resources for research
	Digital networks to knowledge transfer
Marketing	Recruiting new students
	Staying in touch with former students
	Acquiring additional funds
Communication	External communication
	Internal communication
Governance of digital transformation	Responsibility for digital transformation

Source: Almaraz et al. (2016, p. 228)

**Table 5:** Dimentions and levels of digital transformation of universities

Missions	Methodologies and digital tools	Outcomes	Impact (internal and external)	Relations to Q2HM
Teaching	DSI pedagogy, Digital literacy Educating about SI and SDGs New education tools: 3D platforms, virtual simulators Open educational resources like MOOC	New curricula on digital/green challenges DSI integrated in curricula, New digital tools implemented to education New digital and green start-ups and spin offs Open educational repositories New micro and targeted educational programmes	Accommodating different educational needs & integrating vulnerable groups or with diverse backgrounds into university education, Students as “change agents,” good citizens, advocating for human-centric innovation, Equipping society with knowledge relevant to deal with wicked problems Raising “green” awareness and digital literacy	Delivering knowledge and skills related to sustainable priorities and digital challenges and creating power capital
Research	Research on DSI Research creating new SI Research using AI tools	New theories and methodologies in DSI Novel DSI New digital and green start-ups and spin offs Open research repositories	New research networks and research partnerships Growing recognition of solution-oriented research Social impact of research included into the evaluation policy Gap to be addressed due to lack of indicators for measuring impact of DSI and AI research	Establishing new channels for transdisciplinary research on DSI DSI included into regional strategies More inclusive and sustainable innovation ecosystem
Third mission	Public/community/stakeholder engagement in green and digital activities through citizen science Community service Service learning Community-based participatory research (CBPR) Action-research, citizen science, science shops (as methodology) Living labs (as methodology) Promoting DSI through outreach & science communication Social entrepreneurship programmes	Novel DSI New digital and green Community/stakeholder engagement programmes and agendas Novel interactive educational or research platforms open to society	New public–private partnerships for DSI and green deal implementation, New research networks Knowledge democratisation Creating community of practice in DSI Social impact of science included into the evaluation policy	Mutual learning and the new channels of knowledge flow to and from the university allowing for new DSI DSI as part of regional innovation system, New investments in physical, digital, green regional infrastructures on DSI, Regional leadership in DSI
Fourth mission	Universities as open systems in relation to their environment and thus delivering (digital) social innovation, Co-design, Co-creation and Co-delivery for sustainability, DSI as tool for implementing SDGs	Green and digital research and education programmes and curricula focused on sustainable priorities	All missions equally important and mutually enhancing, Green and digital challenges integrated within university strategy and all missions Accountability of universities ensured through appropriate governance and continuous exchange with policy makers, civil society, citizens, business	Opens system of education, research and innovation focused on the environment and sustainability with the use of new digital tools, Continues exchange between university and stakeholders Building regional capacity and resilience though DSI

^*^Only novel approaches or new emphasis in comparison to third mission

Source: Authors

We agree with Procter et al. (2020, p. 5) that since AI is increasingly deployed in academic research in a broad range of disciplines, *steps could be taken to ensure even wider adoption of these new techniques and technologies, including wider training in the necessary skills for effective utilisation of AI, faster routes to culture change and greater multidisciplinary collaboration*. AI represents also a power analytical tool and new digital data resources and become a *“double dividend” for researchers.*

We propose to include the following assumptions in building university ready for Industry 5.0 and Society 5.0. 

Universities should and/or need to:

- Create proper structures and mechanisms supporting the development and implementation of social/digital innovation;
- Extend (digital) social innovation to all the missions;
- Incorporate the societal and sustainability priorities in a systemic way and by this to play an active and leading role in Q2HM;
- Embrace trans- and interdisciplinarity in research and education;
- Promote cross-sector and multi-actor collaboration;
- Incentivise utilisation of AI wherever it can offer benefits to the economy and society;
- Strengthen mobility between industry and academia and recognise other than publications outputs and measures;
- Promote intelligent learning and create new flexible, inclusive, accessible and adaptive learning systems for all generations;
- Promote new curricula focused on green, digital, quantitative and ethical skills necessary to ensure the effective and appropriate utilisation of AI;
- Digital transformation and AI curricula embed in *Responsible Research and Innovation* approach with the aim to anticipate negative impact of AI;
- Focus more on social well-being and the quality of life;
- Deliver tailor-made solutions through social/digital innovation.

They are in line with the current European Universities Association’s vision (European Universities Association, 2021, p.9) of future universities states that:

Europe’s universities will make human-centred innovation their trademark, aiming to achieve sustainability through cooperative models. They will engage in cocreation of solutions with a wide range of partners and with the purpose of meeting common challenges and making a demonstrable difference to society through technological as well as social innovation. As such, universities will play a leading role in innovation ecosystems. They will bring together stakeholders around a common vision, bridging different cultures spanning from academia, business and start-ups, to civil society and the social and cultural scene. They will also reinforce their contribution to the development of knowledge and skills together with partners in the ecosystem.

### Socially and Digitally Engaged Model of University in Society 5.0

The world was already in rapid transformation, but the COVID-19 pandemic has accelerated (amongst others) both green and digital transitions. We need to take advantage of this inflection point so that we do not return to outdated models. Institutions, organisations, companies, universities and all society must transform itself radically and embrace the uncertainty and the transformation “in progress.” We are experiencing and seeking processes rather than definitive solutions. We need a great capacity to update our skills continuously and accept that there are few certainties (Vilalta et al., 2020).

We propose the model of socially and digitally engaged university that embraces new university roles in ecosystem of innovation. In this model, the universities are envisioned as prototyping places for SDT and creating power capital, that is Super Smart Society. We do not focus on AI and other new technologies itself but rather on policies and visions related to the new roles of universities in Industry and Society 5.0 within Q2HM.

**Fig. 5:**
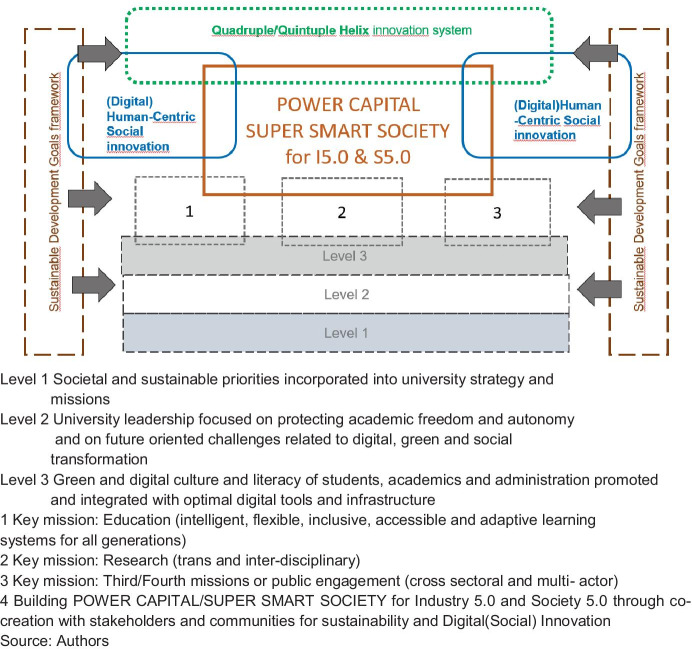
Socially and digitally engaged model of university in Society 5.0

The model is built upon key fundaments and assumptions (Fig. 5). To become more responsible and socially engaged universities new to establish new power relations within university and between science and society. Internally, it means for example to promote and recognise the social activities of students, scientific and administration staff, their public engagement. It also requires promoting organisational culture focused on cooperation, mutual learning, collegiality and the establishment of interdisciplinary task forces. Furthermore, it is necessary to provide a continuous training system related to digital and green transitions and lead an internal information campaign on social innovations so that they become known and understood by the entire academic community. Moreover, if university wants to practice what they preach, they should also promote the culture of equality, equity and anti-discrimination. Externally, it means that university builds relations with stakeholders that rely on the new paradigm of knowledge democratisation reflected by Q2HM in which the knowledge is developed as part of co-construction, i.e., as a result of combining academic and practical knowledge generated by various stakeholders and innovation process participants (Morawska-Jancelewicz, 2021). Universities need more adaptability and flexibility.

The model relies on three fundamental pillars. First, assumes that both societal and sustainable priorities should be incorporated into university strategy and missions. Second, requires a strong leadership protecting the core academic values but also future oriented and understanding the present challenges as part of building power capital. Third, embraces the new green and digital culture and literacy of students that impacts both research, teaching and public engagement. They are linked to basic university missions that incorporate those fundamental assumptions and new university culture. By this, university promotes intelligent, flexible, inclusive, accessible and adaptive learning systems for all generations leading to a new power capital and trans- and interdisciplinary research as well as multi-actor and cross-sectoral public engagement. They are all interrelated and through (digital) human-centric innovation, they create a new innovation ecosystem that is sustainability-oriented and embedded in the Q2HM frameworks. This approach allows universities to contribute more strategically, directly and effectively to present global and local challenges around all the university missions. Still the question “What kind of university do we want for what kind of society?” will not be answered the same in each country (Maasen et al., 2019, p.9). It also reflects the dynamic nature and complexity of present challenges in “adapting to, and adopting, the skills, capacities and capabilities of learning to deal with intellectual uncertainty and of acting and leading beyond the conventional boundaries of disciplinary and professional authority” (Blewitt, 2010, p. 396).

Through (digital) social and human-centric innovations, universities can be a driving force in the sustainable innovation ecosystems encompassing ethical, social, economic and environmental principles. It should rely on trust, sharing and a meaningful sense of identity that will consolidate the network based on shared values, which will enhance sustainable practices (Costa & Matias, 2020, p. 3).

As in the case of firms, the need for *transition towards the digital requires a technological push, and with the acceleration of the innovation processes, universities must be able to quickly identify the value of the open innovation processes, and leverage their competences to speed up the transition to digital* (Costa & Matias, 2020, p. 6). Closely detached to its ecosystems, universities should commit themselves to community needs in the generation of sustainable actions and social welfare through a co-creation process that brings benefits to all the actors involved (Trencher et al., 2013).

## Discussion and Conclusion

One of the main aims of green and digital transitions included in the Industry 5.0 and Society 50 is to ensure that people still lead purpose-driven and creative lives. “*To this end, universities and businesses will have an increasingly crucial role to play. As we move toward a truly people-centric life, progress in information technology must be accompanied by efforts to train up industrial innovators and raise the information literacy of each and every citizen. Universities, for their part, in addition to spurring technological progress as before, must additionally be responsible for cultivating literacy among information users through both general curricula and recurrent education, so as to promote the civil society that embodies Society 5.0*” (Society 5.0 A People-centric Super-smart Society, 2018, p.13).

Universities should take the active role in creating and defining future visions and only responding to them. They need to redefine their roles and the way they act. Some critical questions need to be addressed related to new online/hybrid teaching and educating models responding to novel expectations of different generations. Universities should develop amongst others: adaptive learning programs; collaborative teaching and learning technology and digital resources for lecturers and learners; online learning resources for learners everywhere. They are encourage to develop of curricula which offer students the opportunity to test in practice their competencies and knowledge and to acquire new skills through projects focused on the needs of a specific organisation or local communities like, e.g., service learning (Morawska-Jancelewicz, 2021). Apart from digital, green skills and digital literacy, those new programs need to also teach cognitive and metacognitive skills (critical thinking, creative thinking, self-regulation, etc.); social and emotional skills (empathy, cooperation); and practical and physical skills (using new communication and technology devices, etc.).

Universities should support research of a high social impact and strive for innovations in which nobody claims intellectual property in order to protect it. This opens up ways to social innovation that responds to public and private values and needs. University can respond to social needs through different types of community engagement (living labs approaches, citizen science, science education, including stakeholders in defining their research and education agenda). The digital context of innovation might change the way innovation and knowledge is distributed and created within socio-economic systems. Universities should look for new types of innovation that will be both technical and social. That will help to integrate different approaches through new information and technological channels allowing for including public opinions and voices (e.g., GIS systems in urban development, crowd mapping and crowd sourcing) with social outputs and new types of solutions. The power of new IT tools and AI can lead to more democratic approaches in managing, transferring and distributing the knowledge from and to the society.

Unquestionably, societal values and needs are expressed and codified within the Sustainable Development Goals of UN. They should be reflected in new curricula and research agenda. This globally agreed spectrum of goals asks for urgent actions and solutions developed by different types of stakeholders in co-developed, co-created, co-delivered and co-experimented ways. The Quintuple Helix approach might foster this process as it integrates different perspectives and sets the stage for sustainability priorities and considerations. In this new context of Society and Industry 5.0, the society is at the core of innovation system. Education, research and innovation (E,R,I) are delivered and developed by universities and business which reflect their strong relations in the regional innovation system, and stress the process of lifelong learning that is being continued at work place and stress the need for new paths of flexible learning that should be offered by universities. Those new processes are taking place also in a digital context and it can help to develop new forms and channels of distribution of E,R,I. Human-centric innovation: user-driven innovation, open innovation, (digital) social innovation, empower society and simultaneously engaged them process of the their distribution.

Democracy is a precondition for fully autonomous, free, open future universities. *They need freedom, internally, to pursue their research and teaching and, externally, to engage with society. This requires that universities listen to their communities, recognise the political and social nature and impact of their work, and take responsibility for acting against democratic backsliding* (Jørgensen & Anna-Lena Claeys-Kulik, 2021, p.26). The concept of the Quadruple and Quintuple Innovation Helix framework asserts that an advanced knowledge democracy is actually necessary to advance knowledge and innovation. Educational organisations and higher education institutions (for example, universities) can be expected to play a crucial role. Democracy, environmentalism and the innovation-driven knowledge economy may be co-evolving (Carayannis et al., 2021a, b, c, p. 12).

In this paper, we attempt to address the gap of relatively few studies on institutional change and incentive structures that influence the ability of universities to engage in (digital) social innovation within digital and green transitions. We aim to identify connection between Society 5.0 and Industry 5.0 concepts and the Q2HM framework. We offer a primer on I5.0 and S5.0 by discussing the key definitions and their implicit meanings for the future socially and digitally engaged university model. We can identify few limitations related to our study. First, results directly form the novelty of the Industry 5.0 and Society 5.0 concepts. They are rather future projections but still they represent a wide discussed policy frameworks. Second, consequently, is linked to limited tools and indicators allowing for evaluating and assessing the impact of digital and social innovation. Third is related to the process of green and digital transitions as they need more time to achieve their maturity. Finally, the rapid transformation we face globally, and the complexity and volatility of present challenges (like climate change or political and economic disturbances) might bring further twists that will impact the development of the ecosystems and the role of universities within them. This is on one hand a limitation but it also opens up the new perspectives for further research. We recommend to address the challenge of the twin of green and digital transitions of universities and to showcase the evidence of their alignment and the impact they have on universities missions. Further studies could investigate the practical and evidence-based solutions and implementations of the theoretical assumptions of Industry 5.0 and Society 5.0 (not only within concrete community, location, but also within concrete research, education or outreach activities and projects). This will lead also to addressing the new types of digital and social innovation in innovation ecosystems that integrate human-machines relations and foreground the environmental and sustainable considerations. Since we name those digital and green transitions as disruptions, we also recommend to address the concept of antifragility of universities in further studies and to address both theoretically and empirically the universities responses that go beyond the resilience through co-creation of digital and social innovations.

## Footnotes

1 EUA (2021), *Universities without walls—a vision for 2030, p.10.*
